# Production of Bio-Energy from Pig Manure: A Focus on the Dynamics Change of Four Parameters under Sunlight-Dark Conditions

**DOI:** 10.1371/journal.pone.0126616

**Published:** 2015-05-13

**Authors:** Dongxue Yin, Wei Liu, Ningning Zhai, Yongzhong Feng, Gaihe Yang, Xiaojiao Wang, Xinhui Han

**Affiliations:** 1 College of Forestry and the Research Center of Recycle Agricultural Engineering and Technology of Shaanxi Province, Northwest A&F University, Yangling, Shaanxi, People’s Republic of China; 2 College of Agronomy and the Research Center of Recycle Agricultural Engineering and Technology of Shaanxi Province, Northwest A&F University, Yangling, Shaanxi, People’s Republic of China; Nanyang Technological University, SINGAPORE

## Abstract

This study investigated the effect of sunlight-dark conditions on volatile fatty acids (VFAs), total ammonium nitrogen (TAN), total alkalinity (TA) and pH during pig manure (PM) digestion and then the subsequent influence on biogas yield of PM. PM_1_ and PM_2_ were performed in a transparent reactor and a non-transparent reactor, respectively. Two sets of experiments were conducted with a temperature of 35.0±2.0 °C and a total solid concentration of 8.0% to the digestion material. The dynamic change of the four parameters in response to sunlight-dark conditions resulted in variations of the physiological properties in the digester and affected the cumulative biogas production (CBP). PM_1_ obtained higher CBP (15020.0 mL) with a more stable pH and a lower TAN concentration (1414.5 mg/L) compared to PM_2_ (2675.0 mL and 1670.0 mg/L, respectively). The direct path coefficients and indirect path coefficients between the four parameters and CBP were also analyzed.

## Introduction

With the increasing market demand for pork, the growth of swine herds leads to a large increase in swine manure worldwide [1]. The pollution impact of swine waste on water, soil and air caused is a growing concern in many countries [2, 3]. The sustainability of an efficient disposal mechanism for manure becomes a key factor in the expansion of pig industry in China [4].

Biogas production with PM is a suitable method for the treatment of this organic waste, yielding biogas as a useful by-product. This process could also produce renewable energy (cheap and clean methane), soil conditioner, and liquid fertilizer that are valuable for crop production [2, 5–10]. However, the complex anaerobic digestion processes consisting of a series of microbial reactions are vulnerable to inhibition by many factors, such as sunlight-dark conditions. Recently, a few studies focused on sunlight-dark conditions as an external artificial factor. It was suggested that dark fermentation of organic biomass is a promising technology for producing renewable bio-hydrogen [11, 12]. Research also suggested that bio-hydrogen production by waste materials would be enhanced by sequential dark and light anaerobic fermentations [13]. Rittmann and Herwig and Levin *et al*. showed that dark fermentation can improve the hydrogen evolution rate of bio-hydrogen production and concomitantly produced carbon rich metabolites, like CO_2_ would store in biomass or be converted to other substances, such as CH_4_[14, 15]. Chandra and Mohan suggested that co-culturing photosynthetic bacteria with acidogenic microflora could reduce VFAs accumulation by 40% which could overcome induced fatty acid inhibition during dark-fermentative hydrogen production process [16]. A study by Yin et al. showed that sunlight-dark conditions can increase the biogas yield from PM [3]. However, the promoting influence of sunlight-dark conditions on physiological properties of digester, such as the VFAs, TAN, TA and pH, important parameters to be monitored in anaerobic digestion [17–20], and their effects on biogas production are unclear.

Therefore, the present study emphatically evaluated dynamic changes of the four parameters in the fermentation process of PM under sunlight-dark conditions in order to reveal their effects on biogas production of PM.

## Materials and Methods

### Ethics statement of substrate and inoculum

PM was collected from “Besun Group” swine farm in industrial park, Changqing Road, Yangling, with the permission of the managers. The inoculum was obtained from household biogas digester in 13 North 2nd Street, Cuixigou, which is the model village of biogas utilization and more than 85% households installed biogas digesters. Collection was permitted by the owner Quanyou Cui. Both PM and inoculum were stored in a refrigerator (4.0°C) until use [5]. The experimental procedures were approved by the Ethics Committee of the Research Center of Recycle Agricultural Engineering and Technology of Shaanxi Province in China. Table 1 shows the chemical characteristics of the PM.

**Table 1:** Chemical characterization of substrates used in the digestion experiments.

Material	TS (%)	VS (%)	Organic carbon a (g/kg VS)	Total kjeldahl nitrogen^a^ (g/kg VS)	Carbon-to-nitrogen ratio	pH	TA (mg/L)	TAN (mg/L)	VFAs (mg/L)
PM	27.7	79.2	78.3	6.1	12.8	6.4	5093.0	1328.7	5569.5

^a^ Dry basis

### Experimental design and set-up

**Fig 1:**
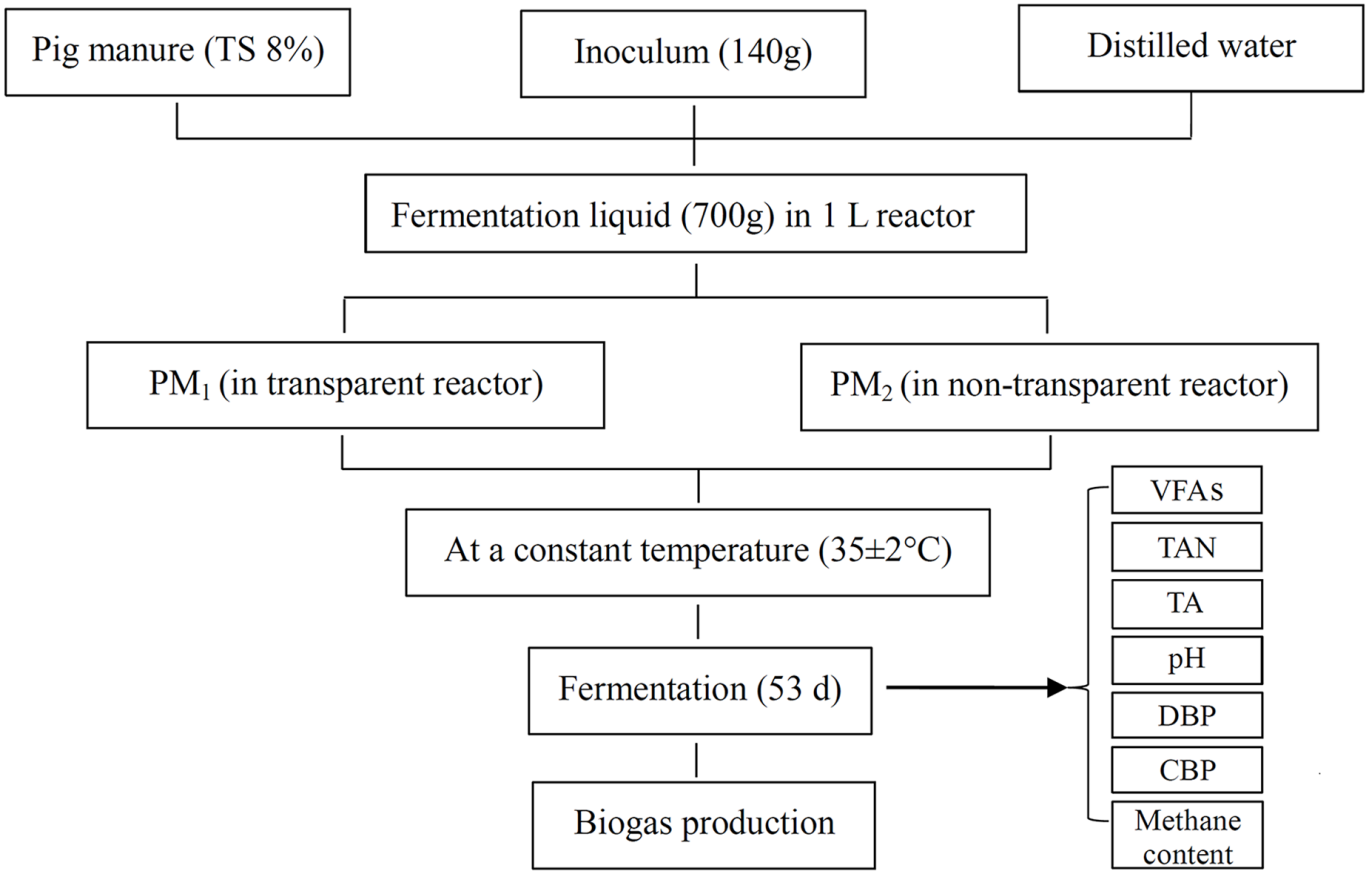
Flowchart of experiment including raw material, the experimental conditions and method.

**Fig 2:**
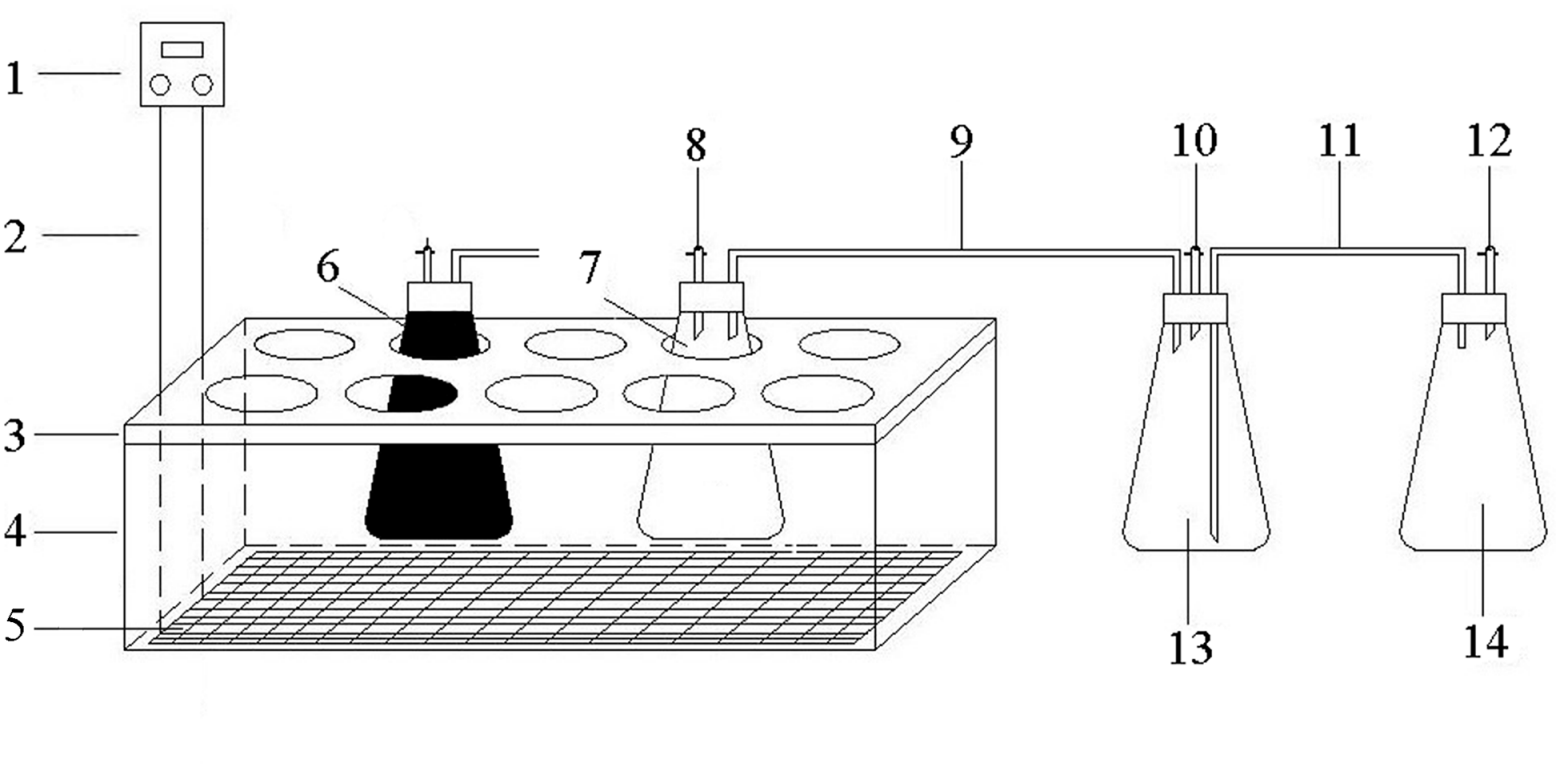
Controlled and constant temperature anaerobic fermentation device. 1. Temperature controlling box; 2. Temperature sensor; 3. Insulated cover; 4. Thermostatic water tank; 5. Strip heater; 6. None transparent digester; 7. Transparent digester; 8. Taking sampling; 9. Airway tube; 10. Taking biogas; 11. Aqueduct; 12. Air pipe; 13. Biogas collecting bottle; 14. Water collecting bottle.

Fig 1 shows the desire of this study. Anaerobic fermentation of PM was carried out in triplicate at 35.0±2.0°C with Total solids (TS) of 8.0% for 53 days. The 1-L digestion reactor with 700.0 g of total liquid, including 140.0 g of inoculum, was conducted under a controlled and constant temperature using an anaerobic fermentation device (Fig 2).

**Fig 3:**
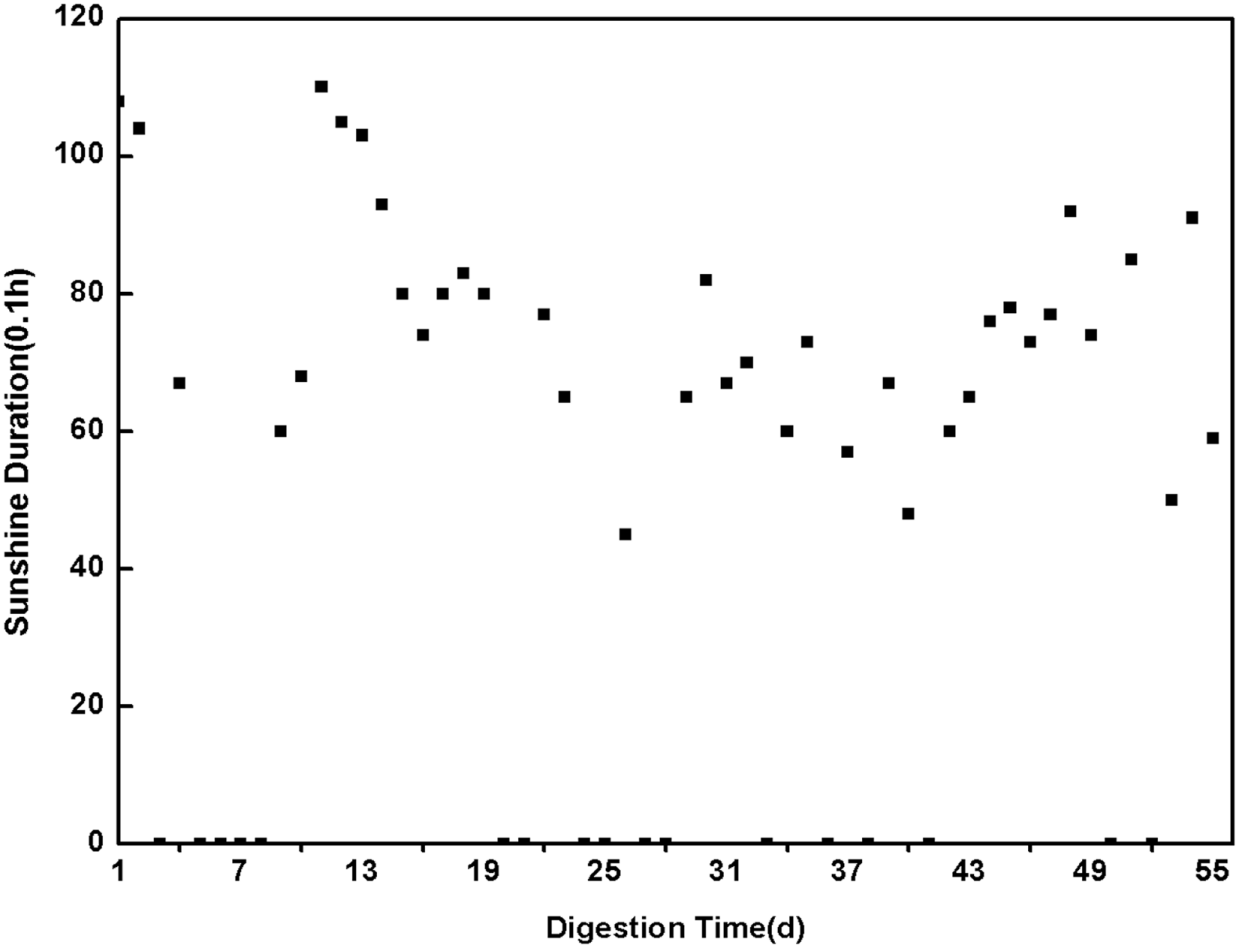
Sunlight duration in Yangling during digestion of PM_1_ and PM_2_.

Two sets of experiments were conducted: one was performed with sunlight-dark fermentation in transparent reactor with nature sunlight (PM_1_), and the other was conducted in total dark in a non-transparent reactor (PM_2_). This work lasted from September 17th to November 11th in 2012. The sunlight duration data (Fig 3) were gathered from the Yangling meteorological information network (http://www.ylqx.gov.cn). The gas volume was measured daily, and the VFA, TAN, TA and pH were measured every 7 days. All fermentation reactors were tested by sealing detection and flushed with nitrogen gas for approximately 3 min to assure anaerobic conditions before measuring [21].

### Analytical techniques

The daily biogas production (DBP) was monitored daily using a drainage gas-collecting method. The content of methane in biogas digester was analyzed by a fast methane analyzer (Model DLGA-1000, Infrared Analyzer, Dafang, Beijing, China). Total organic carbon was determined by the method described in Cuetos *et al*. [22]. The determination of TS and volatile solid (VS) composition was performed according to the APHA Standard Methods [23]. The VFAs concentration was determined using a754P UV spectrophotometer (adding 1.7 mL glycol into 0.5 mL sample before heating for 8 minutes at 90°C; when cooled, transferring this mixed solution to a 25-mL volumetric flask and adding 2.5 mL Hydroxylamine reagent, then diluting with distilled water to volume and mixing it). TAN was analyzed by KDN-08C type semiautomatic azotometer and then titrated with 0.02 N H_2_SO_4_. TA analysis was conducted by titrations with 0.02 M H_2_SO_4_ [18]. pH value was determined by Phs-3ct type pH meters and all titrations were performed in duplicate.

### Statistical analysis

**Fig 4:**
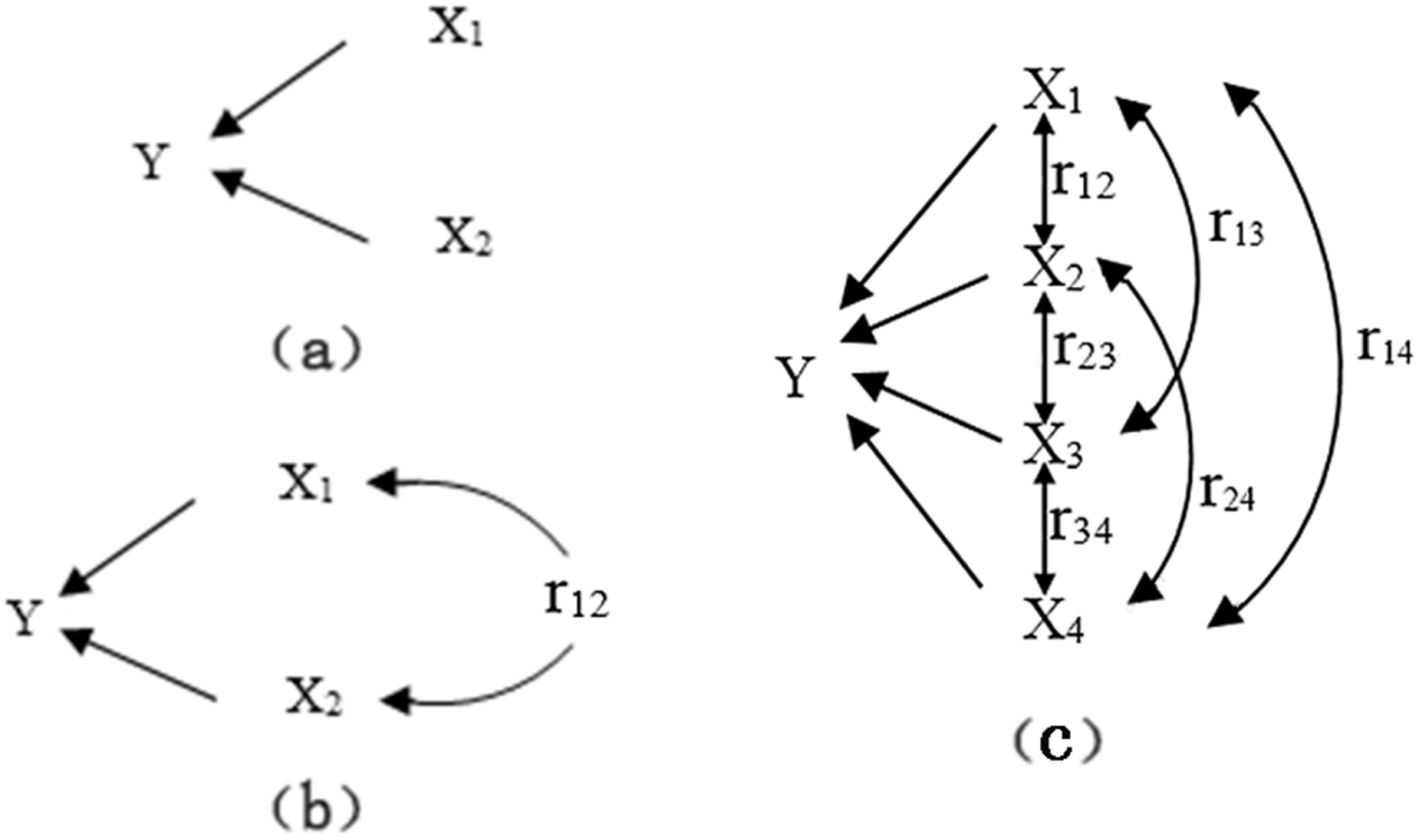
Network to explanation path analysis between independent variables (X_*i*_) and dependent variable(Y). X_1_-VFA; X_2_-TAN; X_3_- total alkalinity; X_4_-pH.

Fig 4 describes path analysis between independent variables (X*i*) and dependent variable (Y). The two arrow lines in Fig 4(a) between the independent variables and dependent variable represent the path where X_1_→Y and X_2_→Y are independent of each other. Fig 4(b) shows four arrow lines that comprise the path network where a correlation exists between X_1_ and X_2_. In addition to the two direct paths (X_1_→Y and X_2_→Y), the path network has two indirect paths attributed to r_12_. One path is generated by the effect of X_1_ on Y via X_2_ (X_1_→X_2_→Y), and another path is generated by the influence of X_2_ on Y via X_1_ (X_2_→X_1_→Y). The above situations can be extended to *p* variables, and the direct path is X_*i*_→Y (*i* = 1, 2…, *p*;). While the indirect path is X_*i*_→X_*j*_→Y (*i*, *j* = 1, 2…, *p; i ≠ j*)[24]. Therefore, the overall effect of X_*i*_ on Y (r_*iy*_)contains two parts: the direct path coefficient (*b*_*i*_) or the direct influence of X_*i*_ on Y (X_*i*_→Y) and the indirect path coefficient (*r*_*ij*_ *b*_*j*_) or the indirect influence of X_*i*_ on Y by X_*j*_ (X_*i*_→X_*j*_→Y, *i* ≠ *j*) (Eq (2)) [24, 25]. Four independent variables were included in our path analysis.

b1+r12b2+…+r1pbp = r1yr21b1+b2+…+r2pbp = r2y ⋮ ⋮ ⋮ rp1b1+rp2b2+…+bp = rpy (1)

$$\;r_{iy}\;=\;b_{i}+\sum_{j\neq 1}b_{j}r_{ij}$$ (2)

Where *b*_*i*_ is the direct path coefficient; *r*_*ij*_ is the correlation coefficient between X_*i*_ and X_*j*_; *r*_*iy*_ is the correlation coefficient between X_*i*_ and Y; and *i*, *j =* 1, 2,*…*, *p*.

## Results and Discussion

### Response of four parameters to sunlight-dark and total dark conditions

#### Dynamic change of VFAs and pH

VFAs, including acetic acid, propionic acid, butyric acid, isobutyric acid, valeric acid, isovaleric acid and n-butyric acid, are organic fatty acids with C_1–6_ and are important intermediary compounds in the metabolic pathway of methane fermentation [26, 27]. In digester, methane bacteria mainly use VFAs to produce methane. However, it does not mean the more VFAs the better, because high concentrations could result in a decrease of pH and increase of non-dissociated fatty acids, which further intensifies inhibition [28, 29]. Therefore, the concentration of VFAs is an important consideration for good performance of a digester. It reflects the imbalance between the microbial groups involved in the degradation. The further degradation of these compounds can proceed only after the removal of hydrogen from the process [30].

**Fig 5:**
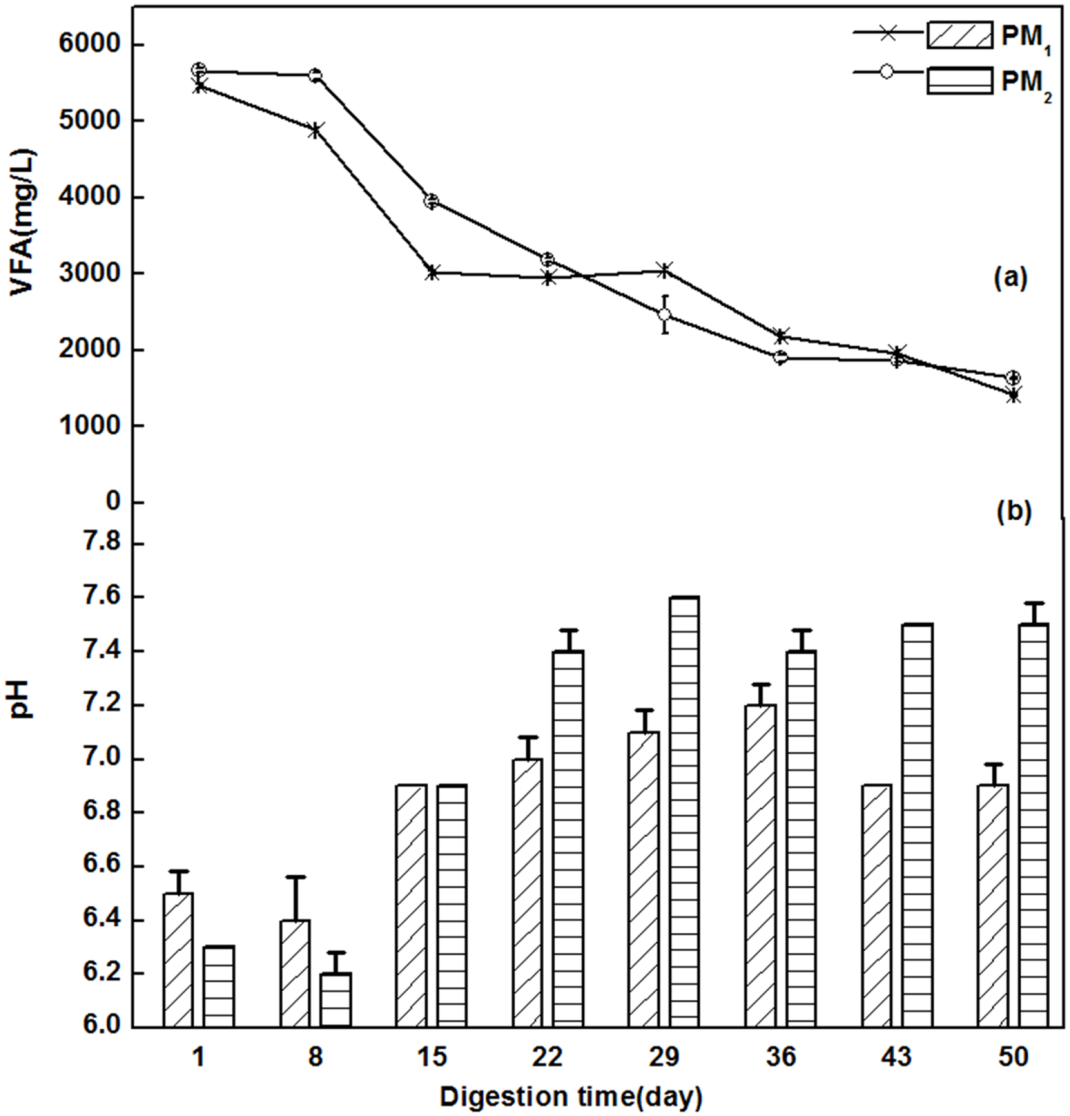
The dynamic changes of VFAs (a) and pH value (b) of PM_1_ and PM_2_.

Fig 5(a) shows dynamic changes of VFAs in PM_1_ and PM_2_. VFAs in PM_1_ and PM_2_ had a decreasing trend in the process of fermentation, which is consistent with the theory of anaerobic fermentation that VFAs were oxidized into substrates slowly by methanogenic bacteria [26]. At the beginning, VFAs decreased sharply, especially in PM_1_, whose VFAs decreased from 5467.5 mg/L to 3018.5 mg/L in the first 15 days of fermentation. After that, the content of VFAs steeply decreased until the 29^th^ day. The final VFAs value was 1418.5 mg/L. The VFAs of PM_2_ had a gentle downtrend in the first 8 days from 5671.5 mg/L to 5598.5 mg/L, and then, this value showed a rapid downward trend until the 36^th^ day of fermentation. Finally, the VFAs in PM_2_ had a similar gradual decrease as that in PM_1_. Comparison the initial and final of fermentation process, the VFAs contents of PM_1_ decreased from 5467.5 mg/L to 1418.5 mg/L and PM_2_ decreased from 5671.5 mg/L to 1633.5 mg/L, respectively.

The stability of the pH in an anaerobic reactor is extremely important because it influences enzymatic activity and the rate of methane production may decrease if the pH is lower than 6.3 or higher than 7.8 [31]. Therefore, a feasible way of improving stability for fermentation would be to monitor and to analyze. As shown in Fig 5(b), on the 8^th^ day of digestion, the pH of PM_1_ and PM_2_ decreased from 6.5 to 6.4 and 6.3 to 6.2, respectively, which could be attributed to hydrolysis acidification. Astals et *al*. showed that large amounts of protein and carbohydrates but small amounts of lipids in PM probably led to hydrolysis acidification [32]. Along with the fermentation process, both sets showed an increasing trend in pH because the acids were rapidly consumed by methanogens, thus increased the pH and stabilized the digester performance [21]. The peak values of PM_1_ and PM_2_ were 7.2 and 7.6 on the 36^th^ and 29^th^ day, respectively. The pH of each group ranged from 6.3 to 7.8 at the end of fermentation and was higher than that of the initial fermentation.

#### Dynamic changes of TAN and TA

Ammonium is an essential nutrient for bacterial growth, but undesirably high concentrations could breakdown the proteins available in the substrate [33]. TAN is also an important parameter influencing methane production by providing buffering capacity [34].

**Fig 6:**
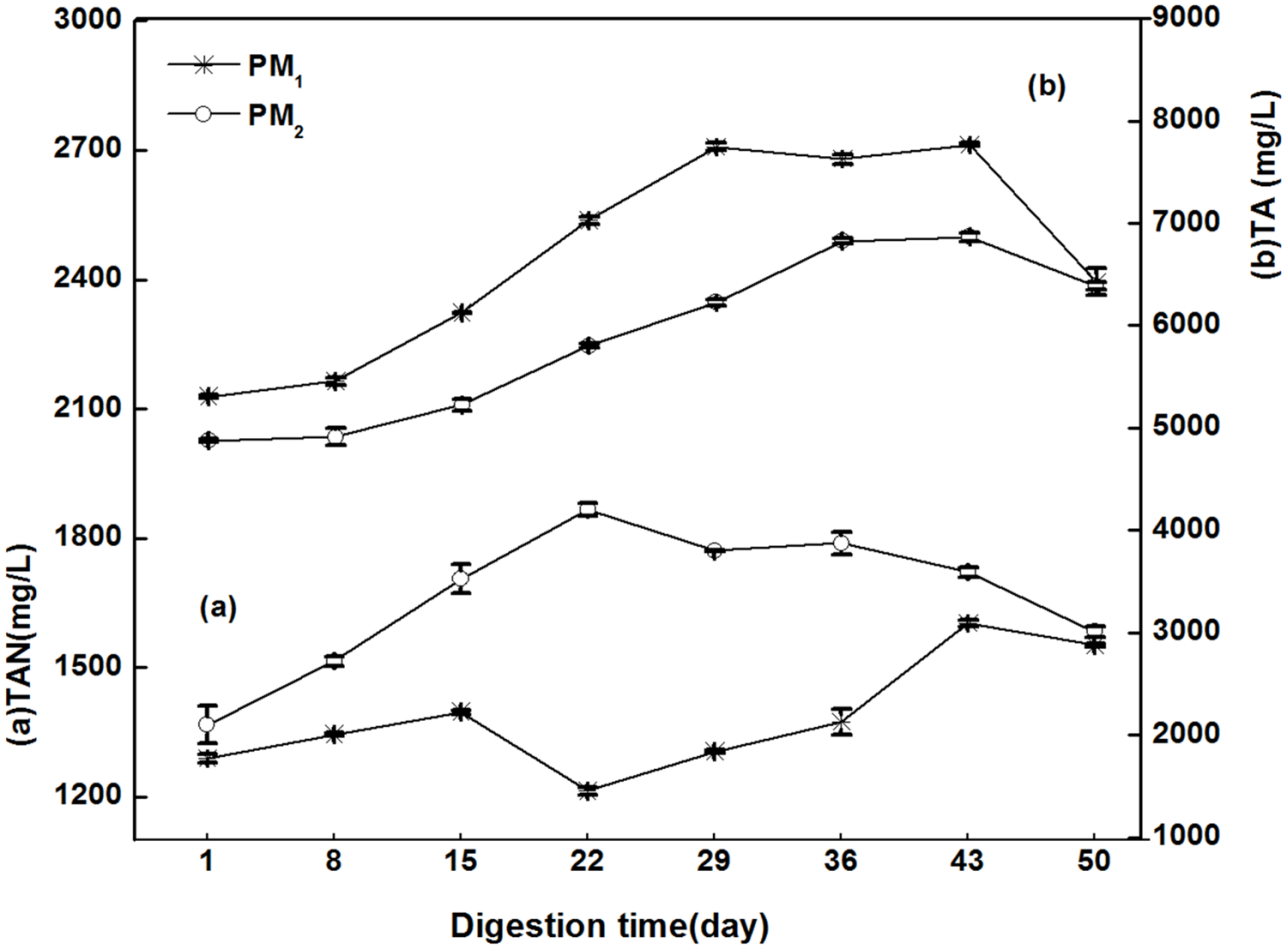
The dynamic changes of TAN (a) and TA (b) of PM_1_ and PM_2_.

Fig 6(a) shows dynamic changes of TAN in PM_1_ and PM_2_. The average amount of TAN in PM_1_ (1385.0 mg/L) was lower than that in PM_2_ (1665.1 mg/L). The value of TAN in PM_1_ increased from 1289.5 mg/L to 1397.2 mg/L and then experienced a slight declined to 1214.1 mg/L on the 22^nd^ day, when the value rebounded and reached a peak of 1602.7 mg/L on the 43^rd^ day. In contrast, PM_2_ had a faster increasing rate of TAN compared to PM_1_ during the first 22 days, increasing from 1367.8 mg/L to a peak value of 1866.5 mg/L, which exceeded the range of 1500.0 mg/L, and the pH reached 7.6. Thus, the biogas production of PM_2_ (2675.0 mL) was significantly less than that of PM_1_ (15020.0 mL). The same conclusion was reported on Calli *et al*. who suggested that ammonia inhibition usually occurs when the pH is above 7.4 and TAN is within the range of 1500.0 mg/L to 3000.0 mg/L [35].

In addition, TA is an ideal parameter to monitor the anaerobic digestion process because of the prevention of pH changes in the reactor and support of buffering capacity [18]. Fig 6(b) shows that TA in PM_1_ and PM_2_ first increased and then decreased until the 43^rd^ day, which probably contributed to the downward trend of VFAs in the digester. The TA value in PM_1_ increased from 5308.0 mg/L to 7746.0 mg/L in the first 29 days of fermentation, then reached a peak of 7766.0 mg/L on the 43^rd^ day, and finally the amount was gradually reduced to 6426.0 mg/L. Similarly to PM_1_, the TA of PM_2_ also peaked at 6866.0 mg/L on the 43^rd^ day, but the peak value of the TA in PM_2_ was less than that of PM_1_ (7766.0 mg/L). Then number decreased to 6386.0 mg/L. As seen in Fig 6(b), the average TA concentration in PM_1_ (6684.8 mg/L) was higher than that of PM_2_ (5891.8 mg/L), but the amount of TA in each group returned to close to the initial value by the end of the experiment.

### Direct and indirect path coefficients between the four parameters and CBP

Path analysis was used to study whether the effects of the four parameters (X_*i*_) on biogas production (Y) are significant and to test the indirect effects of each parameter on CBP by other parameters (X_*i*_→X_*j*_→Y, *i* ≠ *j*). The correlation coefficient (r_*iy*_; Eq (2)) were then obtained. The results of path analysis show that the p-values of the four parameters were significant under different treatment conditions and the p-value of X^pH^_PM1_ and X^pH^_PM2_ were 0.0032 and 0.0026, respectively. According to Eq (2), the direct path coefficients (b_*i*_) added to the indirect path coefficients (r_*ij*_b_*j*_) were equal to the correlation coefficients (r_*iy*_).

Table 2 describes path analysis between VFAs, TAN, TA and pH and CBP of PM_1_ and PM_2_. For PM_1_, X^TA^_PM1_ obtained the maximum b_*i*_ on CBP (-0.6327). However, it had the lowest r_*iy*_ (−0.2190; *p* < 0.05) with CBP because its b_*i*_ was counterbalanced by the r_*ij*_b_*j*_ (0.4137) generated from the interaction among X^TA^_PM1_ and X^VFAs^_PM1_, X^TAN^_PM1_ and X^pH^_PM1_ included in the sum of X^TA^_PM1_ **→**X^VFAs^_PM1_ **→**CBP, X^TA^_PM1_ **→**X^TAN^_PM1_ **→**CBP, X^TA^_PM1_ **→**X^pH^_PM1_ **→**CBP. Under the total dark condition, X^TAN^_PM2_ had the same result as X^TA^_PM1_. X^TAN^_PM2_ achieved the maximum b_i_ values on CBP (0.8355) and the r_*ij*_b_*j*_ value was -0.3621. The value of r_*iy*_ was 0.4714 and lower than that of X^TA^_PM2_ (0.8951) and X^pH^_PM2_ (0.9373). Furthermore, for PM_1_ and PM_2_, the maximum r_*iy*_ was the pH value.

**Table 2:** Path analysis between VFAs, TAN, TA and pH and CBP of PM_1_ and PM_2_.

Parameters	P-value	Direct path coefficients (b_*i*_)	Indirect path coefficients (r_*ij*_b_*j*_)					Correlation coefficients (r_*iy*_)
			X^VFAs^_PM1_	X^TAN^_PM1_	X^TA^_PM1_	X^pH^_PM1_	Total	
X^VFAs^_PM1_	0.0253*	0.2227		-0.2766	0.7613	-0.1395	0.3452	0.5679
X^TAN^_PM1_	0.0056**	0.3417	0.0344		-0.3355	0.583	0.2819	0.6236
X^TA^_PM1_	0.0341*	-0.6327	0.3529	-0.2879		0.3487	0.4137	-0.219
X^pH^_PM1_	0.0032**	0.5163	-0.1286	0.2749	0.1761		0.3224	0.8387
			X^VFAs^_PM2_	X^TAN^_PM2_	X^TA^_PM2_	X^pH^_PM2_	r_*ij*_b_*j*_	r_*iy*_
X^VFAs^_PM2_	0.0456*	-0.5013		-0.1766	0.6796	0.3395	0.8425	0.3412
X^TAN^_PM2_	0.0371*	0.8335	0.0265		-0.2373	-0.1513	-0.3621	0.4714
X^TA^_PM2_	0.0044**	0.4764	0.0344	-0.2355		0.6198	0.4187	0.8951
X^pH^_PM2_	0.0026**	0.7247	-0.0286	0.0651	0.1761		0.2126	0.9373
Correlation coefficients:X^pH^_PM1_ > X^TAN^_PM1_ > X^VFAs^_PM1_> X^TA^_PM1_ X^pH^_PM2_> X^TA^_PM2_> X^TAN^_PM2_ > X^VFAs^_PM2_								

Note:

* P<0.05;

** P<0.01.

The complicated path network relationships between the four parameters and DBP indicate that a single large direct effect (b_*i*_) does not always imply a strong correlation between X_*i*_ and Y. Therefore, further analyses were conducted to take into account the effect of interactions on biogas production and the influence on the physiological properties of the fermentation process [17–20, 36].

### Effects of the four parameters’ dynamic changes and interactions

**Fig 7:**
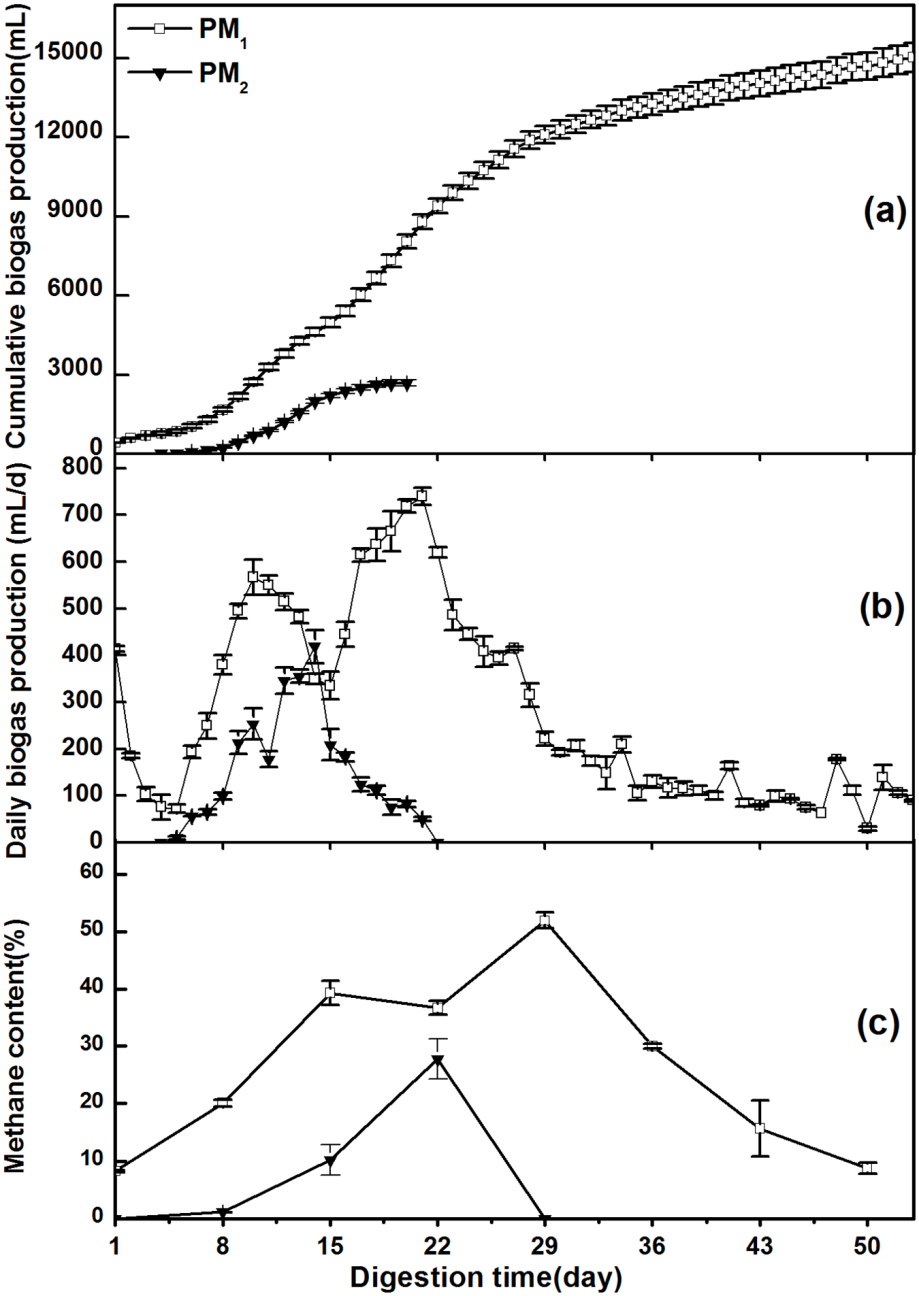
Cumulative biogas production (a), Daily biogas production (b) and Methane content (c) of PM_1_ and PM_2_.

As shown in Table 2 and Fig 5(b), the maximum r_*iy*_ for the two sets was the pH value, but PM_1_ had a similar pH as PM_2_, which was achieved with higher biogas and methane potentials under sunlight-dark conditions. The changes of CBP were shown in Fig 7(a). CBP of PM_1_ during the first half of anaerobic fermentation grew faster than that during the second half, whereas the CBP of PM_2_ gradually increased. The total CBP of PM_1_ (15020.0 mL) was 5.6 times as much as that of PM_2_ (2675.0 mL). This result indicates that the difference of CBP was not due to pH alone. Therefore, further investigation is needed to evaluate the indirect effects of different parameters on biogas production.

Fig 7(b) shows that the DBP of PM_2_ was lower than that of PM_1_ and resulted in reduced methanogen reactiveness, which was caused by higher average accumulations of VFAs in PM_2_ (3286.4 mg/L) and higher amount of TAN in PM_2_ (1866.5 mg/L) with a pH 7.4 on the 22^nd^ day. Calliet *et al*. explained that ammonia inhibition usually occurs when the pH is above 7.4 and TAN is within the range of 1500.0 mg/L to 3000.0 mg/L [35]. Moreover, hydrolysis acidification easily occurs in PM digestion that has large amounts of protein and carbohydrates and low levels of lipids. Thus, the low average total alkalinity in PM_2_ (5891.0 mg/L) resulted in a low buffer capacity and reduced ability to prevent the acidification of fermentation [37]. According to Table 2, the r_*iy*_ between VFAs and CBP for PM_2_ had a minimum value of 0.3412 but the indirect effect generated by VFAs→TA→CBP (X^VFAs^_PM2_ **→**X^TA^_PM2_ **→**CBP) reached 0.6796 and the indirect effect generated by TAN→pH→CBP (X^TAN^_PM2_ **→**X^pH^_PM2_ **→**CBP) reached 0.1513, which may be plausible reasons for the shorter fermentation time and lower biogas production in PM_2_ than in PM_1_. Along with fermentation, the DBP of PM_1_ and PM_2_ gradually increased and peaked with increasing pH levels and the r_*iy*_ between pH and DBP was largest. The maximum DBP of PM_1_ was 740.0 mL/d on the 21^st^ day, whereas that of PM_2_ was 419 mL/d on the 14^th^ day. The maximal biogas yield occurred at a pH of 6.5 to 7.5[38], which is consistent with the findings of the current study. After the peak, the DBP began to slide.

Fig 7(c) shows the changes of CH_4_ content. PM_1_ had higher CH_4_ potentials than PM_2_. PM_1_ showed the highest methane content of 51.9% on the 29^th^ day, followed by a sharp decrease to 8.8% at the end of the experiment. This trend confirmed the change of DBP in PM_1_ (Fig 7(b)). For PM_2_, the low biogas yield in the short fermentation time (20 days) caused the CH_4_ content to be lower than that of PM_2_ and rapidly dropped after reaching the maximum value (27.8%) on the 22^nd^ day.

## Conclusion

The differences in four parameters caused by sunlight-dark conditions significantly affected the CBP. PM_1_ achieved 15020.0 mL of CBP, which was 5.6 times as much as PM_2_. Direct (X_*i*_ **→**Y) and indirect effects (X_*i*_ **→**X_*j*_→Y) among four parameters on CBP determined the values of r_i*y*_ that, which were different in PM_1_ (X^pH^_PM1_ > X^TAN^_PM1_ > X^VFAs^_PM1_> X^TA^_PM1_) and PM_2_ (X^pH^_PM2_> X^TA^_PM2_> X^TAN^_PM2_ > X^VFAs^_PM2_). It was suggested that the dynamic change of pH had the most dramatic effect on the fermentation performance of PM_1_ and PM_2_ and TA and VFA had the weakest influence on the fermentation performance of PM_1_ and PM_2_, respectively.
